# Effect of Coconut Shell and Zinc-Modified Biochar Composite Applications on Phthalate Ester (PAE) Accumulation, Growth Performance, and Soil Health in Wheat (*Triticum aestivum* L.)

**DOI:** 10.3390/toxics14090810

**Published:** 2026-09-11

**Authors:** Aisha Khaled Chaudhri, Aansa Rukya Saleem, Nazneen Bangash, Abdulaziz Alamri, Mostafa A. Abdel-Maksoud, Aljawharah Fahad Alabbad, Habib Ullah

**Affiliations:** 1 Department of Earth and Environmental Sciences, School of Engineering and Applied Sciences, Bahria University, Islamabad 44000, Pakistan; aishachaudhri1@gmail.com; 2 Centre of Research Excellence, Earth, Climate and Environemnt (CORE-ECE), Bahria University, Islamabad 44000, Pakistan; 3 Department of Biosciences, COMSATS University Islamabad, Islamabad 44000, Pakistan; nazneen.bangash@gmail.com; 4 Biochemistry Department, College of Science, King Saud University, P.O. Box 2455, Riyadh 11451, Saudi Arabia; abalamri@ksu.edu.sa; 5 Research Chair of Biomedical Applications of Nanomaterials, Biochemistry Department, College of Science, King Saud University, P.O. Box 2455, Riyadh 11451, Saudi Arabia; mabdmaksoud@ksu.edu.sa; 6 Botany and Microbiology Department, College of Science, King Saud University, P.O. Box 2455, Riyadh 11451, Saudi Arabia; alalabbad@ksu.edu.sa; 7 School of Intelligent Electrical and New Energy, Qingdao Hengxing University of Science and Technology, Qingdao 266042, China; 8 Innovation Center of Yangtze River Delta, Zhejiang University, Jiaxing 314100, China

**Keywords:** zinc-modified biochar, phthalate esters (PAEs), DMP, DBP, *Triticum aestivum* L., soil remediation, e-waste contamination, adsorption kinetics, soil fertility, sustainable agriculture

## Abstract

Soil contamination with phthalate esters (PAEs), particularly in e-waste-impacted regions, poses serious threats to crop productivity, soil health, and environmental sustainability. This study investigates the effectiveness of coconut shell biochar (BC) and zinc-modified biochar (Zn-BC) in mitigating PAE accumulation, improving wheat (*Triticum aestivum* L.) growth, and restoring soil fertility in contaminated soils. Characterization analyses revealed that Zn-BC possessed enhanced surface functionality, crystallinity, and elemental composition, favoring greater adsorption potential. Batch adsorption experiments demonstrated that Zn-BC exhibited higher equilibrium adsorption capacities for DMP (23.9 mg/g) and DBP (26.6 mg/g), following pseudo-second-order kinetics. Zn-BC significantly improved plant growth parameters including shoot height, root length, and biomass compared to unamended soil in a pot experiment. Post-harvest soil analysis showed increased pH (6.6), CEC (18.3 cmolc/kg), TOC (1.85%), and microbial biomass carbon (320 mg/kg) under Zn-BC treatment. Enzymatic activities such as dehydrogenase (45.2 µg TPF/g/d) and phosphatase (58.6 µg PNP/g/h) also improved markedly, indicating restored biological activity. These findings highlight the potential of Zn-BC as a sustainable amendment for remediating phthalate-contaminated soils and improving crop health under environmental stress conditions.

## 1. Introduction

The rapid growth of electronic waste (e-waste) has emerged as a significant environmental concern due to the uncontrolled disposal and recycling practices that release a wide range of hazardous pollutants into the environment. Among these, phthalate esters (PAEs), such as dimethyl phthalate (DMP) and di-n-butyl phthalate (DBP), are widely used plasticizers that have been frequently detected in soils surrounding informal e-waste recycling sites. Electronic waste (e-waste), defined as discarded electrical and electronic equipment such as computers, mobile phones, and circuit boards, has emerged as a rapidly growing environmental pollutant [1]. These persistent endocrine-disrupting compounds impose serious risks to soil health, crop productivity, and human well-being through food chain contamination. Consistently with this concern, exposure to other endocrine-disrupting agrochemical contaminants has been shown to disrupt the hypothalamic–pituitary–ovarian axis and hormone homeostasis in mammalian models, underscoring the broader physiological risks posed by such persistent pollutants [2]. In the present study, soil health is considered in a functional ecological context, referring to the capacity of soil to perform key ecological functions, including supporting plant productivity, regulating nutrient cycling, sustaining microbial and biochemical processes, and buffering or regulating the mobility and bioavailability of contaminants. This functional interpretation is consistent with the broader concept of soil-ecological functions described in classical soil-ecological frameworks, including that developed by Dobrovolsky and co-workers [3] which considers soil as a multifunctional component of terrestrial ecosystems and emphasizes its diverse roles in sustaining ecosystem processes and environmental quality. The physicochemical and biological parameters measured in the present study—including pH, cation-exchange capacity (CEC), total organic carbon (TOC), nutrient status, microbial biomass carbon, and soil enzyme activities—were therefore used as indicators related to these functions rather than as a comprehensive assessment of all soil-ecological functions. It is important to note that soil response to polychemical contamination varies significantly depending on soil type, texture, organic matter content, and mineral composition. The environmental behavior of PAEs is governed by interactions between contaminant-specific properties and the soil matrix. Variations in soil organic carbon, clay and mineral composition, pH, cation-exchange characteristics, and pore-water chemistry can modify sorption–desorption equilibrium and consequently regulate the mobility and bioavailability of PAEs. Therefore, differences in these properties may produce contrasting contaminant retention and exposure even among soils subjected to similar external PAE inputs [4].

Wheat (*Triticum aestivum* L.) is one of the most important staple cereal crops globally, and particularly in Pakistan, as it contributes significantly to food security and the agricultural economy. However, in regions exposed to environmental pollution, wheat cultivation is increasingly threatened by soil contaminated with phthalate esters (PAEs), such as dimethyl phthalate (DMP) and di-n-butyl phthalate (DBP) [4]. Once accumulated in soil, PAEs are taken up by plants, suppressing growth, development, and yield quality. Warm and humid conditions prevalent in some agricultural areas may further influence the mobility and bioavailability of these contaminants, exacerbating their adverse effects on crop performance [5].

Biochar has gained attention as a promising soil amendment due to its high surface area, porosity, and ability to immobilize organic pollutants while improving soil fertility [6]. Previous studies have demonstrated that biochar can adsorb PAEs, although adsorption performance varies considerably with feedstock, pyrolysis conditions, surface modification, and PAE molecular properties. For example, graphene-oxide-modified biochar exhibited adsorption capacities of 35.2, 26.4, and 25.1 mg g^−1^ for DMP, DEP, and DBP, respectively, with the dominant interactions differing among individual PAEs [7]. Similarly, adsorption of DBP onto fresh and oxidized corncob biochar ranged from approximately 9.86–13.2 mg g^−1^ under single-solute conditions, while oxidation enhanced PAE adsorption through changes in surface interactions. These findings indicate that modification of biochar can alter its affinity for PAEs; however, the magnitude and mechanism of enhancement depend on both the modification strategy and the physicochemical characteristics of the target PAE [6]. PAE retention by biochar can involve several concurrent physicochemical interactions. The porous carbonaceous matrix provides sites for pore filling and hydrophobic partitioning, while aromatic domains may contribute to π–π interactions with the aromatic ring of phthalate molecules. Oxygen-containing surface functional groups can additionally participate in hydrogen-bonding interactions. Metal functionalization may further alter surface charge, functional-group chemistry, porosity, and the availability of additional surface interaction sites. However, because post-adsorption spectroscopic characterization was not performed in the present study, these interactions are considered plausible mechanisms rather than experimentally confirmed individual adsorption pathways. Coconut shell-derived biochar offers an environmentally friendly, low-cost, and carbon-rich solution [8]. Nevertheless, the adsorption behavior of phthalate esters (PAEs) on unmodified biochar is compound- and matrix-dependent, reflecting differences in molecular structure, polarity, hydrophobicity, and the physicochemical properties of the sorbent and surrounding medium [9]. For instance, previous studies report that pristine biochar archives only 40–60% removal of DBP from aqueous solutions compared to 85–95% removal by metal-modified counterparts however. However, these values are system-specific and should not be generalized across different PAEs, sorbents, or environmental matrices [10].

Compared to the other established remediation techniques for PAE-contaminated soils, Zn-modified biochar may offer potential advantages by combining contaminant immobilization with amendment-related improvements in soil properties. However, its effectiveness and environmental safety depend on the amendment dose and soil physicochemical characteristics, particularly pH, texture, organic carbon content, mineral composition, cation-exchange capacity, and the resulting bioavailability of Zn and target contaminants. Comparable dose- and matrix-dependence has been reported for other metal-immobilization soil remediation strategies, such as magnesium phosphate cement solidification of copper-contaminated soil, where engineering and environmental performance likewise depended strongly on the treatment dose and native soil properties [11]. Phytoremediation using plants such as Ryegrass or Mustard can uptake and degrade PAEs but is slow (often requiring multiple growing seasons), temperature-dependent, and limited by plant tolerance to high pollutant loads [12]. Chemical oxidation using Fenton reagents or persulfates achieves rapid PAE degradation but is costly, may generate toxic by-products, and can disrupt soil microbial communities and structure [13]. Bioremediation using PAE-degrading bacteria (e.g., Bacillus and Rhodococcus) is environmentally friendly but often fails in field conditions due to competition with native microbes, nutrient limitations, and low bioavailability of more hydrophobic PAEs like DBP [14]. Beyond biological and biochar-based approaches, other porous adsorbent materials such as zeolites have also been widely explored for pollutant capture owing to their tunable porosity and surface chemistry, offering a further point of comparison for adsorption-based remediation strategies [12].

Similarly, the adsorption capacity of unmodified biochar for DMP is often below 20 mg/g, whereas the modified biochar’s can exceed 30 mg/g [13]. To overcome these limitations, chemical modifications have been explored. Metal-modification biochar, particularly zinc-modified biochar (Zn-BC), demonstrate improved contaminant immobilization and nutrient cycling due to the introduction of active sites and synergistic effects between metal oxides and carbonaceous matrices [14]. Zinc is an essential micronutrient involved in several plant enzymatic and physiological processes; however, its beneficial effects are concentration-dependent, and excessive bioavailable Zn may adversely affect plant growth and soil biological processes [15]. Despite increasing interest in biochar-based remediation of PAE-contaminated soils, several knowledge gaps remain. First, much of the available adsorption research has examined individual PAE compounds under simplified aqueous conditions, whereas e-waste-impacted soils may contain mixtures of PAEs and other co-contaminants within complex soil matrices. Second, although metal functionalization can modify the surface chemistry and sorption behavior of biochar, the specific contribution of Zn functionalization to the retention of structurally different PAEs such as DMP and DBP requires further investigation. Third, relatively limited attention has been given to the simultaneous effects of Zn-modified biochar on plant growth and soil biochemical indicators, including soil enzyme activities, under PAE-contaminated conditions [16]. Therefore, integrated evaluation under contaminated-soil conditions is needed to determine whether Zn functionalization improves PAE immobilization while maintaining beneficial effects on soil and plant health. We hypothesized that Zn functionalization of coconut-shell biochar would modify its surface and sorption characteristics, resulting in greater immobilization of DMP and DBP than pristine biochar. We further hypothesized that this enhanced immobilization, together with amendment-induced changes in the soil environment, would alleviate PAE-associated stress on wheat and improve physicochemical and biological indicators related to soil functioning.

This study addresses the following research questions:

(i) Can Zn-modified biochar enhance immobilization of DMP and DBP compared to pristine biochar?
(ii) How does Zn-BC affect wheat growth and physiological performance under PAE stress?
(iii) Can Zn-BC restore soil fertility and microbial activity in e-waste contaminated soils?

The experimental scope was limited to a single composite contaminated surface/rhizosphere soil, one biochar application rate (2%, *w*/*w*), and controlled pot conditions. Consequently, the study was not designed to resolve site-specific soil-type effects, individual pedogenic-horizon responses, amendment dose–response relationships, or long-term field-scale effects.

## 2. Materials and Methods

### 2.1. Study Area and Soil Sampling

The study was conducted in the Islamabad–Rawalpindi metropolitan region, Pakistan (approximately 33.5–33.9° N, 72.9–73.2° E; 469–540 m above sea level), which has a subtropical climate and predominantly sandy-loam agricultural soils [17]. A total of 14 soil samples were collected from the 0–15 cm wheat (*Triticum aestivum* L.) root zone at peri-urban agricultural locations affected by anthropogenic activities, including informal e-waste handling, roadside deposition, and disposal of electronic and plastic waste near irrigation channels. No formal source-apportionment analysis was performed; therefore, detected PAEs were not attributed exclusively to e-waste sources.

Sampling locations included Khanpur (KP), Bani Gala (BG), Alipur (AP), Qasim Farm (QF), NCRD Islamabad (NCRD), Shehzad Farm (SF), Sanam Chowk (SC), Dokazak (D), Commodore School (CS), Farash Town (FT), Chak Shahzad Islamabad (IC), Golra Islamabad (IG), Khyaban-e-Kashmir (IKK), and Rawalpindi Road (R) (Table 1). At each location, five subsamples were collected within a 10 × 10 m^2^ grid and composited into one representative sample (~2 kg per location). The 0–15 cm depth was selected to represent the actively cultivated wheat root zone [15].

### 2.2. Chemicals

The DMP (≥99.0%), DBP (≥99.8%), and chromatographic-grade methanol (≥99.0%) were obtained from Thermo Fisher Scientific Co., Ltd. (Shanghai, China). Urea (≥99.5%), zinc sulphate heptahydrate (ZnSO_4_·7H_2_O, ≥99.0%), potassium dihydrogen phosphate (KH_2_PO_4_, ≥99.5%), and potassium chloride (KCl, ≥99.0%) were obtained from Sigma-Aldrich (St. Louis, MO, USA). Stock solutions of DMP and DBP (1000 mg L^−1^) were prepared separately in methanol and stored in amber glass bottles at 4 °C [15]. For batch adsorption experiments, 25 mg L^−1^ working solutions were freshly prepared by dilution with deionized water and protected from light when not in use [18].

### 2.3. Soil Preparation and Baseline Characterization

#### 2.3.1. Sample Pretreatment and Composite Soil Preparation

Following transport to the laboratory, soil samples were air-dried at 25 ± 2 °C, sieved through a 2 mm stainless-steel mesh, and homogenized. Equal masses of the 14 site-level composites were combined, with each site contributing 1/14 of the final composite soil by mass, and thoroughly mixed to obtain homogeneous soil for the pot experiment. A portion of each sieved sample was stored at −20 °C for subsequent physicochemical and PAE analyses [19].

#### 2.3.2. Initial Physicochemical Properties of Composite Soil

Prior to the pot experiment, the composite soil was characterized for baseline physicochemical properties using standard protocols. Soil pH was measured in a 1:5 soil-to-water suspension using a calibrated pH meter, and electrical conductivity (EC) was determined in the corresponding soil–water extract using an EC meter. Total organic carbon (TOC) was determined by the Walkley–Black wet oxidation method, total nitrogen (TN) by Kjeldahl digestion, distillation, and titration, and cation exchange capacity (CEC) using neutral 1 M ammonium acetate extraction. Soil texture was determined by the hydrometer method based on particle sedimentation according to Stokes’ law. The resulting baseline properties are presented in Table 2.

#### 2.3.3. PAE Analysis of Composite Soil

Initial DMP and DBP concentrations in the composite soil were determined by gas chromatography–mass spectrometry (GC–MS) following ultrasonic extraction with dichloromethane according to USEPA Method 3550C. Total PAE concentration was calculated as the sum of DMP and DBP concentrations. The measured concentrations are presented in Table 2.

### 2.4. Biochar Production and Zinc Modification

#### 2.4.1. Feedstock Preparation

Coconut shells were collected from local street vendors in Rawalpindi and Islamabad. The shells were washed with deionized water and then sun-dried for 72 h until constant weight. The dried shells were mechanically crushed into small pieces (1–2 cm) using a stainless-steel grinder, followed by fine powder preparation using a laboratory ball mill (PM 100, Retsch, Haan, Germany) at 300 rpm for 30 min. The resulting powder was sieved through a 0.5 mm mesh to ensure uniform particle size.

#### 2.4.2. Pyrolysis

The powdered coconut shell biomass was subjected to pyrolysis in a muffle furnace (Nabertherm GambH, Lilienthal, Germany) under oxygen-limited conditions. The temperature was ramped from room temperature to 600 °C at a heating rate of 10 °C min^−1^ and held at 600 °C for a residence time of 2 h [20]. After pyrolysis, the biochar was allowed to cool to room temperature inside the furnace under a continuous nitrogen flow (0.5 L min^−1^). The resulting biochar was gently ground and sieved through a 0.25 mm mesh.

#### 2.4.3. Zinc Modification Through Impregnation Method

**Figure 1:**
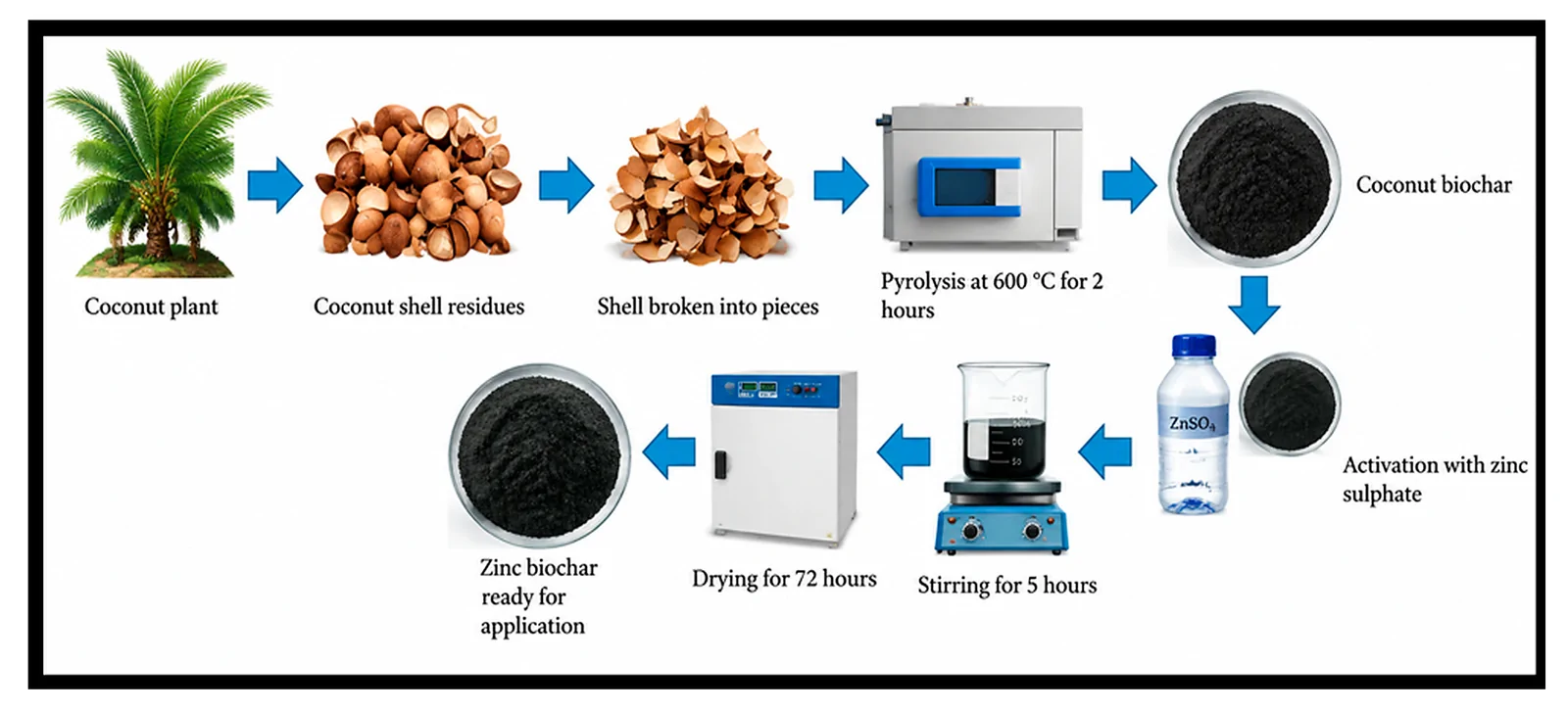
Schematic representation of biochar production and zinc modification: coconut shell collection, biochar synthesis and modification with zinc sulphate.

Zinc-modified biochar (Zn-BC) was prepared using the wet impregnation method described by Marcinczyk with some modification [18]. A ZnSO_4_. 7H_2_O solution (1250 mg L^−1^ Zn^2+^) was prepared in deionized water. Biochar (BC) was added to the ZnSO_4_ solution at a solid-to-liquid ratio of 1:10 (*w*/*v*) (i.e., 10 g of biochar per 100 mL of ZnSO_4_ solution). The mixture was placed in an orbital shaker and agitated at 150 rpm for 5 h at room temperature (25 ± 2 °C) to ensure uniform impregnation. After shaking, the suspension was vacuum filtered through Whatman No. 42 filter paper [21]. The impregnated biochar was then oven-dried at 105 °C for 12 h until constant weight. The dried Zn-BC was gently ground and sieved through a 0.25 mm mesh. Both unmodified biochar (BC) and zinc-modified biochar (Zn-BC) were stored in airtight glass containers at room temperature in a desiccator until further use (Figure 1).

#### 2.4.4. Yield Calculation

The biochar yield was calculated using the formula:

Yield (%) = (Mass of biochar after pyrolysis/Mass of dry feedstock) × 100(1)

The measured yield of unmodified BC was 28%, while that of Zn-BC after modification was 26.5%.

### 2.5. Characterization

Physicochemical properties of BC and Zn-BC (pH, EC, moisture, ash, and yield) were determined following standard protocols. Surface morphology and elemental composition were analyzed by scanning electron microscopy coupled with energy-dispersive X-ray spectroscopy (SEM-EDX) using a JEOL JSM-IT200 scanning electron microscope (JEOL Ltd., Tokyo, Japan) equipped with an Oxford Instruments EDX detector. Samples were gold-coated before analysis. SEM was performed at an accelerating voltage of 15 kV and a working distance of approximately 10 mm, with images acquired at magnifications of 500×–5000×.

Elemental composition was further determined by X-ray fluorescence spectroscopy (XRF) using an Axios FAST XRF spectrometer (PANalytical, Almelo, the Netherlands). Samples were oven-dried and homogenized before analysis.

Surface functional groups were identified by Fourier-transform infrared spectroscopy (FTIR) using a PerkinElmer Spectrum Two (Waltham, MA, USA) over 400–4000 cm^−1^ at a resolution of 4 cm^−1^ using the KBr pellet method. Crystalline structure and mineral phases were analyzed by X-ray diffraction (XRD) using a Bruker D8 Advance diffractometer with Cu Kα radiation (λ = 1.5406 Å), operated at 40 kV and 40 mA. Diffraction patterns were recorded over a 2θ range of 10–80° at a scanning rate of 2° min^−1^ [22]. Average crystallite size (D) was estimated using the Scherrer equation, D = Kλ/(β cos θ), where K = 0.9, λ = 1.5406 Å, β is the full width at half maximum (FWHM, radians), and θ is the Bragg diffraction angle [23].

### 2.6. Batch Adsorption Experiment of DMP and DBP

Batch adsorption experiments were conducted in triplicate (*n* = 3) to evaluate DMP and DBP adsorption by BC and Zn-BC. For each experiment, 0.5 g of BC or Zn-BC was added to 100 mL of individual DMP or DBP solution (25 mg L^−1^) in 250 mL amber glass bottles. The suspensions were shaken at 150 rpm and 25 ± 1 °C. Aliquots (5 mL) were collected at 0, 5, 15, 30, 60, 90, and 120 min and immediately filtered through 0.45 µm PTFE syringe filters.

Residual DMP and DBP concentrations were determined by UV–visible spectrophotometry at 274 and 280 nm, respectively [24]. Calibration curves were prepared using DMP and DBP standards ranging from 0 to 50 mg L^−1^. The amount adsorbed at time *t*, q_t_ (mg g^−1^), was calculated as:

q_t_ = (C_0_ − C_t_) × V/m (2)

C_0_ and C_t_ (mg L^−1^) are the initial and residual phthalate concentrations at time “t”, V (L) is the solution volume, and “m” (g) is the adsorbent mass. Adsorption kinetics were evaluated using pseudo-first-order and pseudo-second-order models: Pseudo-first-order model:

dq/dt = k1 (qe − qt)(3)

Pseudo-second-order model:

dq/dt = k^2^ (qe − qt)^2^ (4)

where:

dq/dt = rate of change in absorbed amount with time;

qt = amount of adsorbate adsorbed at time t (mg g^−1^);

qe = adsorption capacity at equilibrium (mg g^−1^);

k1 = pseudo-first-order rate constant (min^−1^);

t = contact time (min).

Kinetic parameters were determined by nonlinear regression analysis using OriginPro 2021 (OriginLab Corporation, Northampton, MA, USA).

### 2.7. Pot Experiment Design

#### 2.7.1. Experimental Treatments and Biochar Application Rate

The pot experiment consisted of three treatments with three replicates each (*n* = 3), arranged in a completely randomized design (CRD): (i) control (CK), contaminated soil without biochar; (ii) BC, contaminated soil amended with unmodified coconut shell biochar; and (iii) Zn-BC, contaminated soil amended with Zn-modified coconut shell biochar. BC and Zn-BC were applied at 2% (*w*/*w*), equivalent to 20 g of biochar per 1 kg of soil, based on previous studies [25], and thoroughly mixed with the soil.

#### 2.7.2. Soil Preparation and Contamination Level

The composite contaminated soil described in Section 2.3 was used without additional DMP or DBP spiking. Initial concentrations were 12.4 ± 1.8 mg kg^−1^ DMP and 18.7 ± 2.3 mg kg^−1^ DBP (Table 2) [26].

#### 2.7.3. Pot Preparation and Incubation

Each plastic pot (volume of 1 dm^3^, height of 8 cm, circumference of 45 cm) was filled with 1 kg of treated or untreated soil, resulting in a soil depth of approximately 6 cm. Pots were spaced approximately 5 cm apart and maintained at 60% water-holding capacity (WHC) with deionized water. Before sowing, the soils were incubated in the dark at 16–18 °C for 55 days. Soil moisture was maintained at approximately 60% WHC by weighing individual pots every second day and replacing the recorded water loss with deionized water [27].

#### 2.7.4. Sowing and Thinning

After incubation, eight winter wheat seeds [28] were sown per pot at a depth of 1 cm. Seven days after germination, seedlings were thinned to five uniform plants per pot [28].

#### 2.7.5. Growth Conditions

Pots were transferred to a controlled growth chamber under a 16 h light/8 h dark photoperiod, day/night temperatures of 26–28/16–18 °C, relative humidity of 60–70%, and light intensity of 300 µmol m^−2^ s^−1^ provided by fluorescent lamps [29]. Soil moisture was maintained at 60% WHC throughout the 55-day growth period, and no additional fertilizers were applied. Plants were harvested 55 days after sowing [30].

### 2.8. Plant Growth Measurements

At harvest (55 days after sowing), shoot height, root length, and fresh biomass were measured [31]. Shoot height (cm) was measured from the soil surface to the tip of the tallest leaf using a measuring tape. Roots were carefully washed with deionized water, and root length (cm) was measured from the root crown to the longest root tip. For fresh biomass (g plant^−1^), plants were separated into shoots and roots, gently blotted dry with filter paper, and weighed using an analytical balance (precision ± 0.01 g). Measurements were performed for each treatment (*n* = 3), and results were expressed as mean ± standard deviation [32].

### 2.9. Soil Fertility and Health Assessment

Post-harvest soil samples were collected from each pot, air-dried at 25 ± 2 °C for 48 h, and sieved through a 2 mm mesh. All analyses were performed in triplicate (*n* = 3). Soil pH and electrical conductivity (EC) were measured in a 1:5 (*w*/*v*) soil-to-deionized-water extract using calibrated pH and EC meters, respectively [33]. Cation exchange capacity (CEC) was determined using the ammonium acetate method (pH 7.0) by leaching soil with 1 M NH_4_OAc and quantifying displaced NH_4_^+^ by Kjeldahl distillation [34]. Total organic carbon (TOC) was determined using the Walkley–Black dichromate oxidation method [34], and total nitrogen (TN) by the Kjeldahl digestion method [35]. Nitrate (NO_3_^−^) and ammonium (NH_4_^+^) were extracted with 2 M KCl at a 1:10 soil-to-solution ratio and quantified using a continuous-flow auto-analyzer [36].

Microbial biomass carbon (MBC) was determined by the chloroform fumigation-extraction method [32]. Fumigated and non-fumigated soil samples (10 g each) were extracted with 0.5 M K_2_SO_4_, and organic carbon in the extracts was measured using a total organic carbon analyzer. MBC was calculated as [37]:

MBC = (C fumigated − C non-fumigated)/0.45(5)

Dehydrogenase activity was determined by colorimetric measurement of triphenyl formazan (TPF) produced from 2, 3, 5-triphenyl tetrazolium chloride (TTC). Soil (5 g) was incubated with TTC solution at 37 °C for 24 h; the resulting TPF was extracted with acetone and measured at 485 nm. Results were expressed as µg TPF g^−1^ d^−1^ [34]. Phosphatase activity was determined using p-nitrophenyl phosphate (PNP) as substrate. Soil (1 g) was incubated with 0.1 M PNP solution at 37 °C for 1 h, and released p-nitrophenol was measured at 410 nm. Results were expressed as µg PNP g^−1^ h^−1^ [38].

### 2.10. Statistical Analysis

The pot experiment comprised three treatments (CK, BC, and Zn-BC) with three biological replicates per treatment (*n* = 3). Treatment effects on each response variable were evaluated using one-way analysis of variance (ANOVA). Normality and homogeneity of variance were assessed using the Shapiro–Wilk and Levene’s tests, respectively. When significant treatment effects were detected, Tukey’s HSD post hoc test was applied for pairwise comparisons at *p* < 0.05. Statistical analyses were performed using SPSS version 23.0 (IBM Corp., Armonk, NY, USA), and results are presented as mean ± standard deviation (SD). Adsorption kinetic data were analyzed separately by nonlinear regression and were not included in the ANOVA-based multiple-comparison analysis.

## 3. Results

### 3.1. Characterization of Biochar and Zinc-Modified Biochar

#### 3.1.1. Physical Properties and Textural Characteristics

The physicochemical and textural properties of unmodified biochar (BC) and zinc-modified biochar (Zn-BC) are presented in Table 3. The measured pH values were 6.5 ± 0.1 for BC and 6.8 ± 0.1 for Zn-BC, while EC values were 2.1 ± 0.2 and 2.6 ± 0.2 µS cm^−1^, respectively. The measured yields of BC and Zn-BC were 28.0 ± 1.5% and 26.5 ± 1.2%, respectively. Zn-BC exhibited a higher BET surface area (267.8 ± 6.1 m^2^ g^−1^) and total pore volume (0.19 ± 0.01 cm^3^ g^−1^) than BC (185.4 ± 5.2 m^2^ g^−1^ and 0.12 ± 0.01 cm^3^ g^−1^, respectively), whereas the average pore diameters were 3.6 ± 0.2 and 3.8 ± 0.2 nm, respectively.

#### 3.1.2. Surface Morphology and Elemental Composition

**Figure 2:**
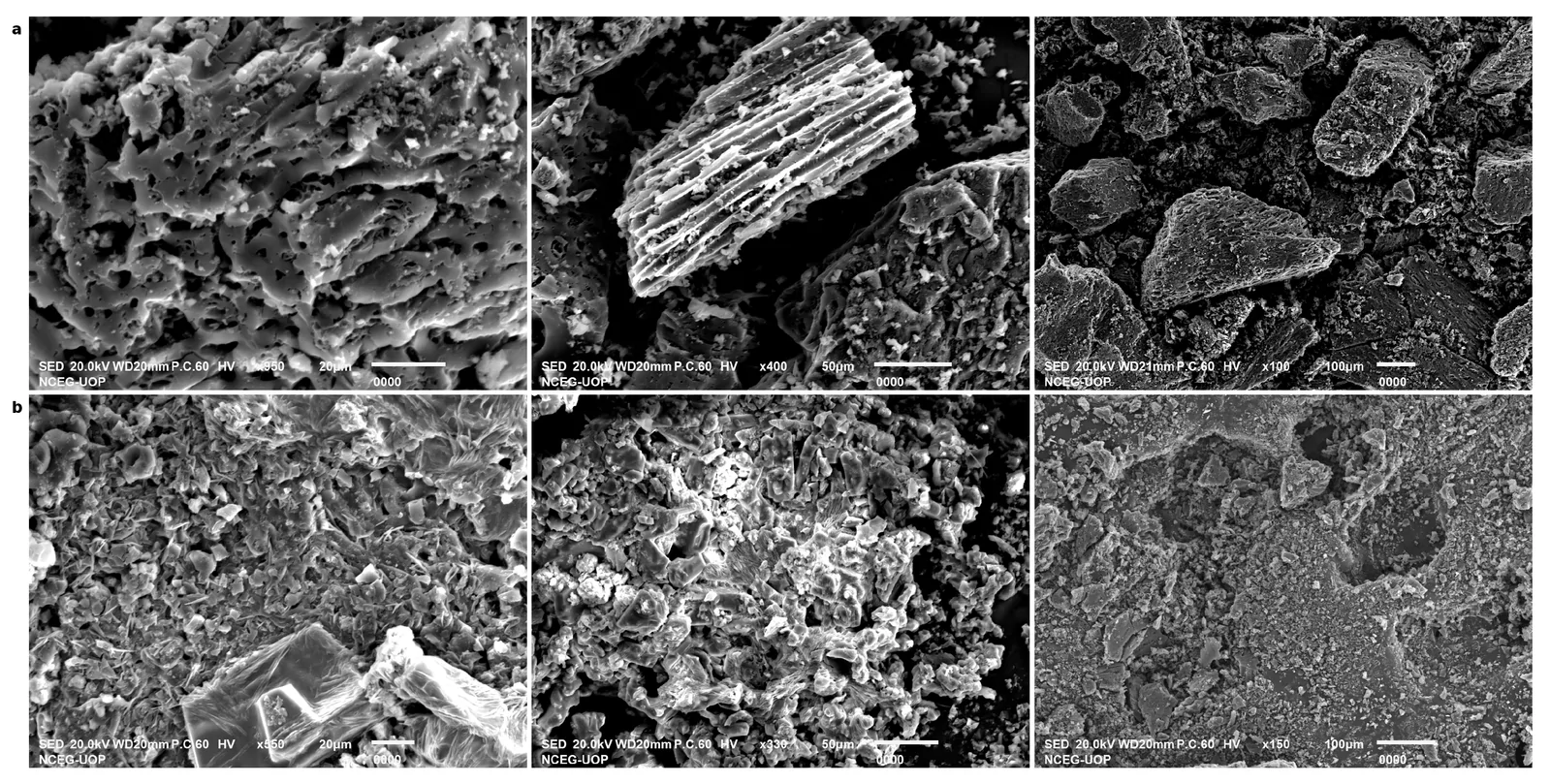
SEM images of biochar (**a**) and zinc-modified biochar (**b**) at different magnification scales: 20 µm, 50 µm and 100 µm.

**Figure 3:**
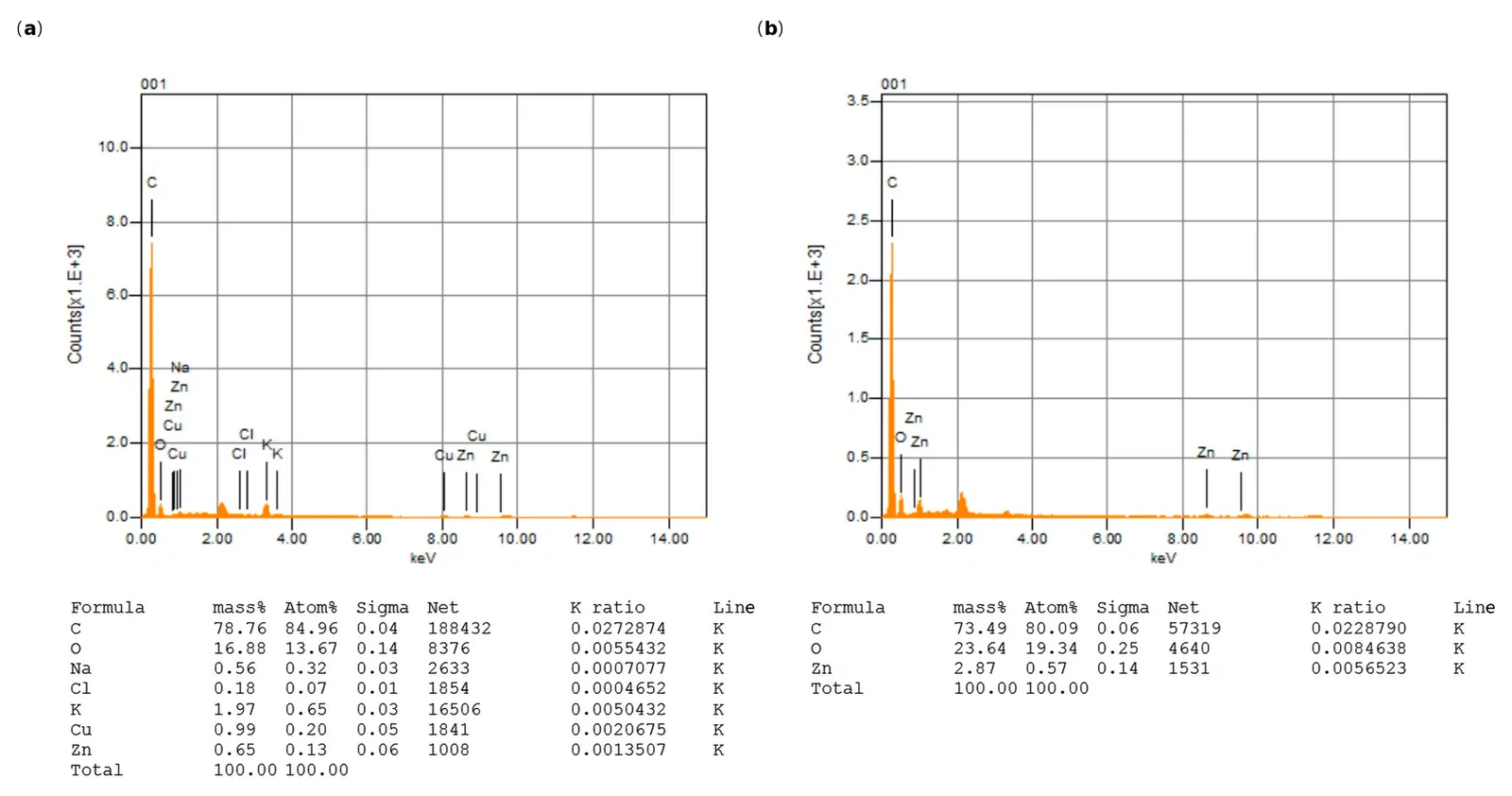
SEM-EDS spectra of (**a**) biochar and (**b**) zinc-modified biochar.

SEM-EDX analysis revealed that Zn modification increased surface porosity and the abundance of light spots (Figure 2), suggesting higher ash and oxygen content, both favorable for adsorption. Zn-BC exhibited enhanced surface roughness with visible Zn-based nanoparticles deposited on the biochar matrix. X-ray fluorescence (XRF) analysis provides quantitative elemental composition data for both biochars (Table 4). Zn modification resulted in a substantial increase in Zn content from 120 ppm (BC) to 8138 ppm (Zn-BC), confirming successful impregnation. Notably, Fe and Mn contents were also increased by 22.6% and 25.0%, respectively, likely due to the ZnSO_4_ solution extracting trace metals from the biochar or impurities in the modification reagent. These elevated transition metal concentrations may contribute to enhanced catalytic and redox potential for organic pollutant degradation (Figure 2 and Figure 3) [39].

#### 3.1.3. Functional Groups (FTIR)

**Figure 4:**
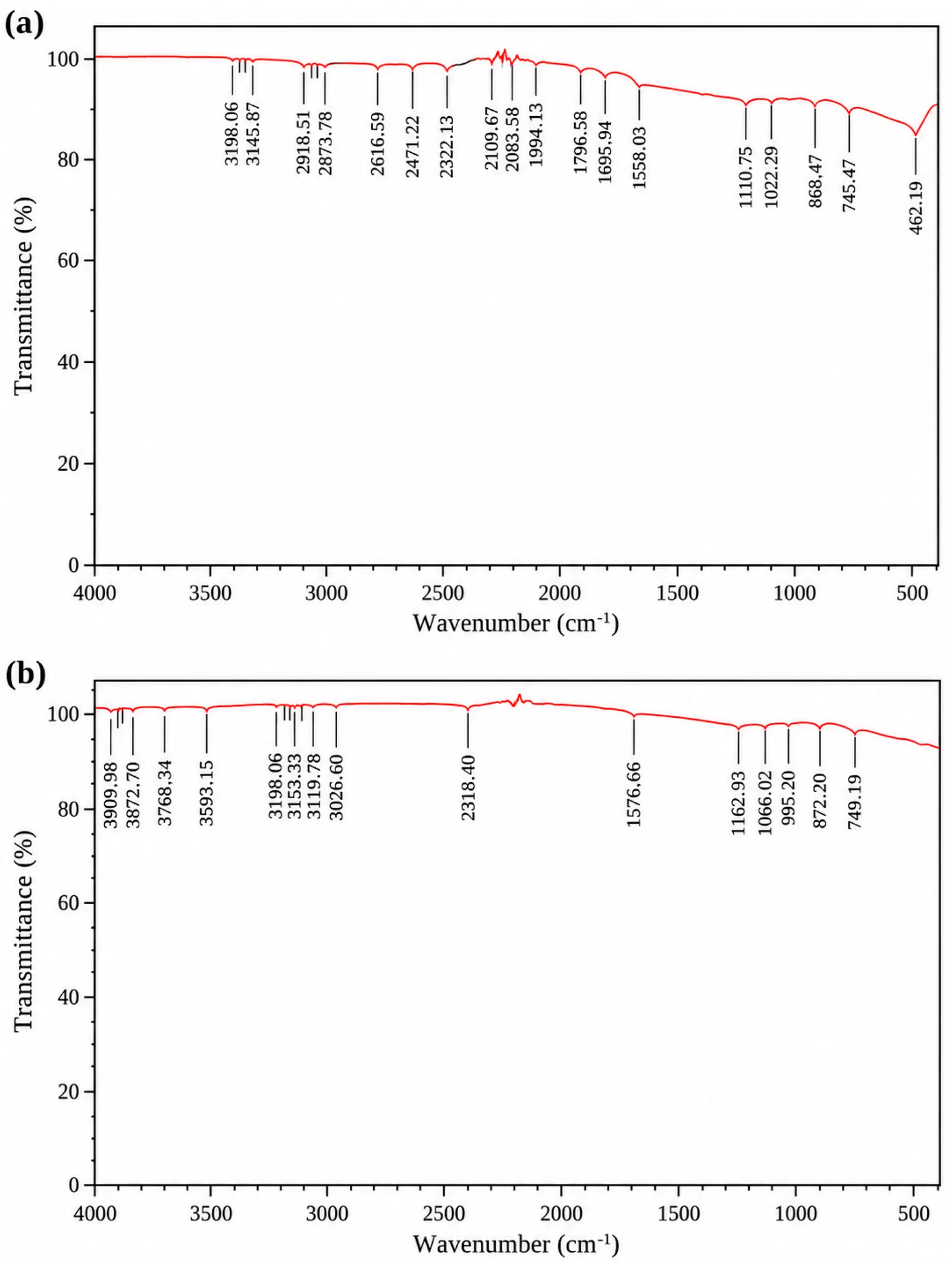
FTIR spectra of (**a**) biochar and (**b**) zinc-modified biochar.

The FTIR spectra of BC and Zn-BC are presented in Figure 4. BC exhibited bands at 3198.1 and 3145.9 cm^−1^ in the O–H stretching region, while corresponding bands were observed around 3198.1 cm^−1^ in Zn-BC. A band at 1769.6 cm^−1^ in BC was assigned to C=O stretching of oxygen-containing functional groups. Additional prominent bands were observed at 1550.8, 1110.7 and 1021.7 cm^−1^ for BC and at 1576.8, 1162.9 and 1060.9 cm^−1^ for Zn-BC. Zn-BC also exhibited a band at 2318.4 cm^−1^. Because FTIR was employed for qualitative functional-group characterization, differences in peak intensities or transmittance between BC and Zn-BC were not subjected to statistical comparison.

#### 3.1.4. Crystallinity (XRD)

**Figure 5:**
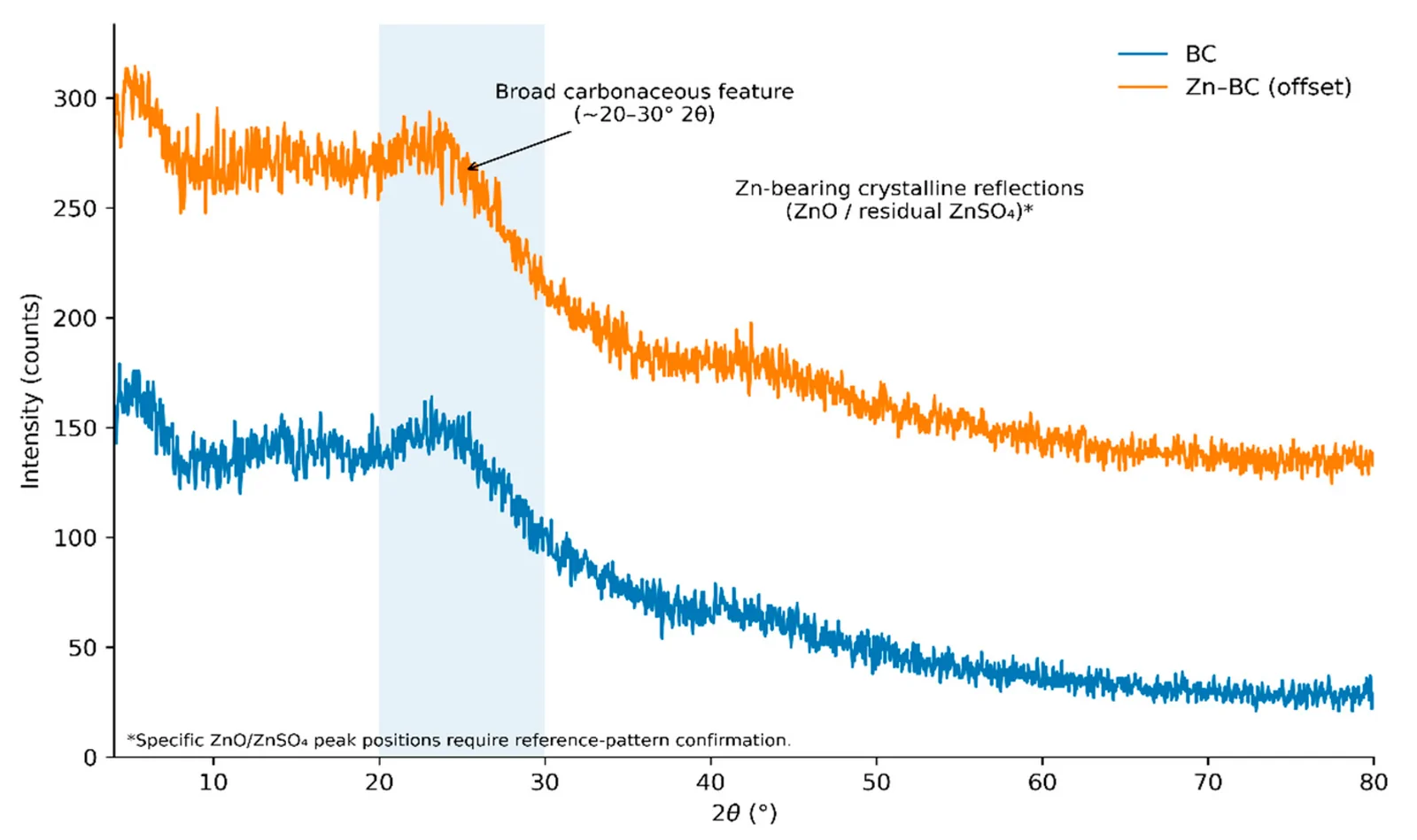
XRD spectra of biochar (blue) and zinc-modified biochar (orange). The light-blue shaded region highlights the broad carbonaceous diffraction feature at approximately 20–30° 2θ. Both materials exhibited a broad diffraction feature at approximately 20–30° 2θ, characteristic of the predominantly disordered carbonaceous structure of biochar. Additional crystalline reflections in Zn-BC were attributed to Zn-containing phases, including ZnO and residual ZnSO_4_, indicating the presence of mixed Zn-bearing crystalline phases following modification. The Zn-BC pattern is vertically offset for clarity.

XRD patterns of BC and Zn-BC are presented in Figure 5. Zn-BC exhibited additional crystalline reflections attributable to Zn-containing phases, including ZnO and residual ZnSO_4_ derived from the ZnSO_4_ precursor used during modification. The presence of ZnSO_4_-related reflections indicates that the deposited Zn phase was not exclusively ZnO and that a fraction of the precursor remained in the modified biochar. The average crystallite sizes estimated using the Scherrer equation were 169.39 nm for BC and 252.19 nm for Zn-BC. Accordingly, the Zn-BC material is described as containing mixed Zn-bearing crystalline phases rather than phase-pure ZnO. (Figure 5). Both BC and Zn-BC exhibited a broad diffraction feature in the approximately 20–30° 2θ region, consistent with the predominantly disordered carbonaceous structure of biochar. Owing to the broad and poorly resolved nature of this feature, a reliable quantitative comparison of graphitic peak position and corresponding d-spacing between BC and Zn-BC could not be established. Therefore, changes in aromatic stacking and associated π–π interactions with PAEs were not inferred quantitatively from the present XRD data.

### 3.2. Batch Adsorption of DMP and DBP

The higher adsorption capacity of Zn-BC compared with BC may be associated with differences in its physicochemical and surface characteristics. Potential interactions involved in PAE retention may include hydrophobic interactions, hydrogen bonding, π–π interactions, and interactions with surface functional groups. However, these mechanisms were not directly investigated using post-adsorption spectroscopic analysis and are therefore considered plausible interactions rather than experimentally confirmed adsorption mechanisms. The adsorption data were better described by the pseudo-second-order (PSO) kinetic model, with R^2^ values of approximately 0.999 (Table 5). However, the superior fit of the PSO model is interpreted as a mathematical description of the adsorption kinetics and not as direct evidence of chemisorption.

The adsorption data were better described by the pseudo-second-order (PSO) kinetic model (R^2^ ≈ 0.999); however, the superior PSO fit was interpreted as a mathematical description of the adsorption kinetics rather than direct evidence of chemisorption. Zn-BC exhibited a higher equilibrium adsorption capacity (q_e_) than BC for both PAEs. The q_e_ values for DMP were 21.40 and 23.90 mg g^−1^ for BC and Zn-BC, respectively, whereas those for DBP were 22.19 and 26.64 mg g^−1^, respectively.

### 3.3. Pot Experiment: Plant Biometric and Soil Fertility

**Figure 6:**
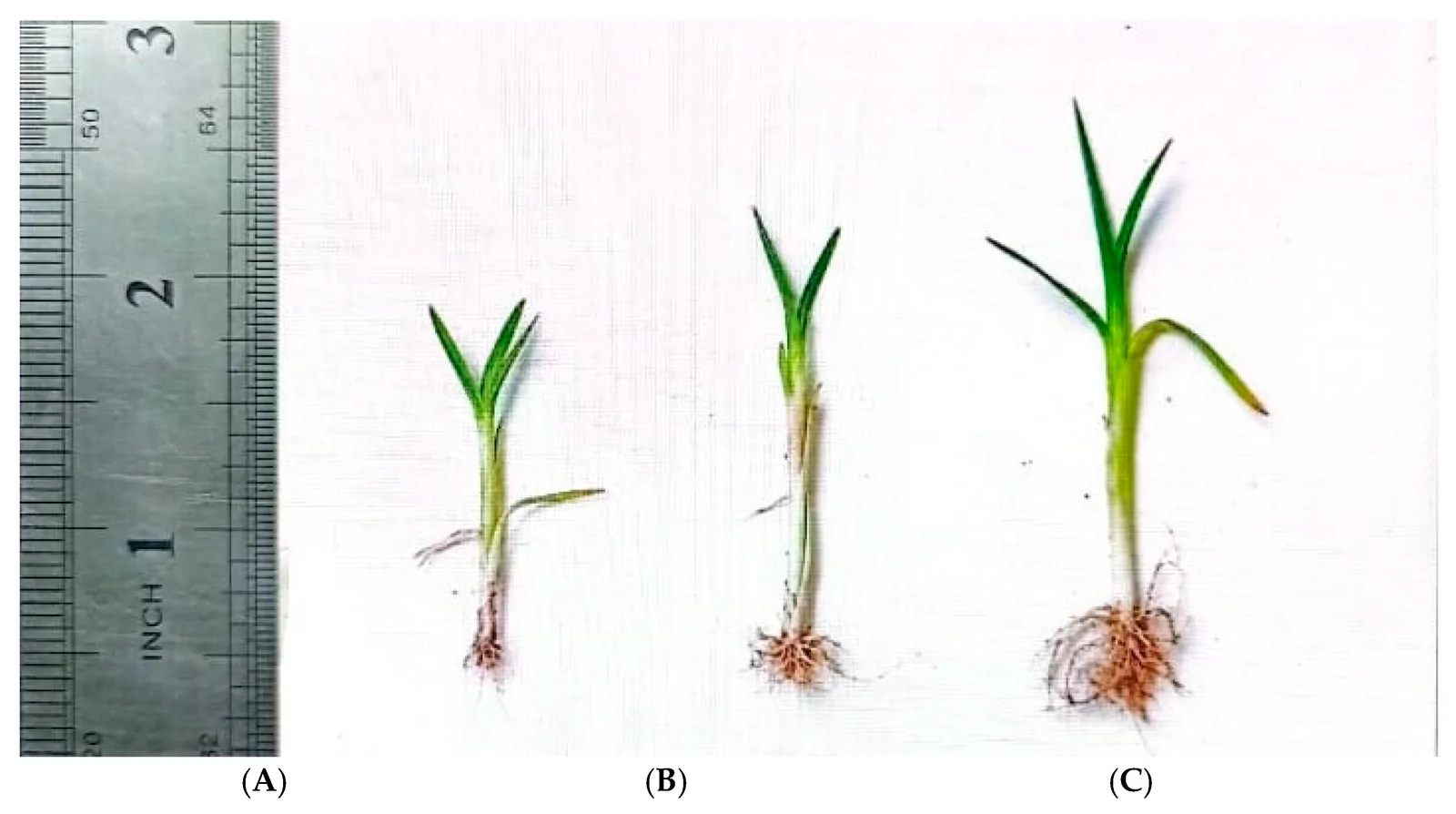
Growth of wheat plants under different treatments. (**A**) Whole plant under control; (**B**) whole plant under biochar application; (**C**) whole plant under zinc-modified biochar. Mean shoot length at harvest was 17.40 ± 0.10 cm (control), 23.20 ± 0.10 cm (BC), and 29.43 ± 2.70 cm (Zn-BC) (Table 6), providing a quantitative size reference for the visual comparison shown.

**Figure 7:**
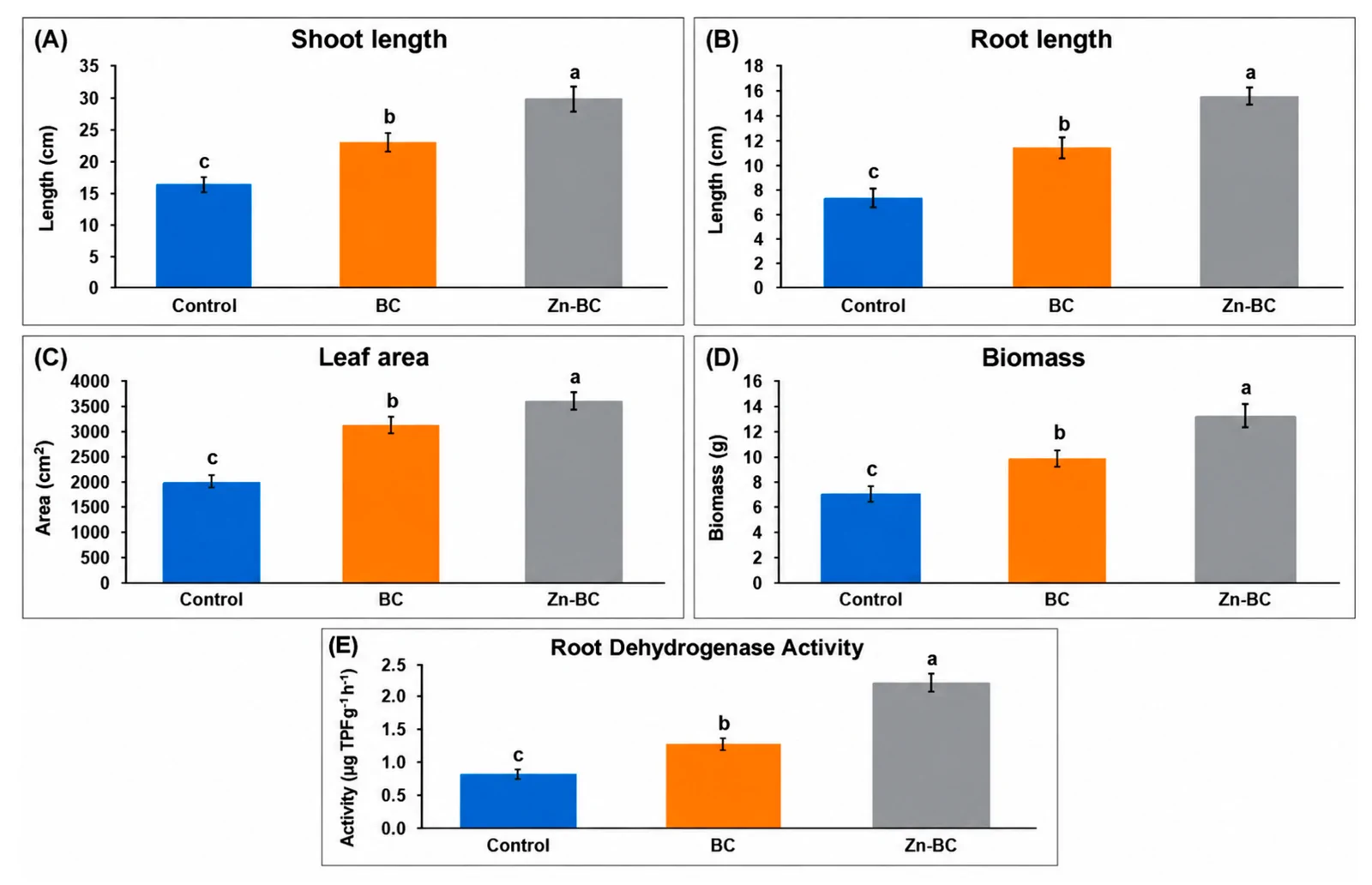
Effects of biochar (BC) and zinc-modified biochar (Zn-BC) on plant growth attributes and soil dehydrogenase activity under experimental conditions. Panels represent (**A**) shoot length, (**B**) root length, (**C**) biomass accumulation, (**D**) leaf area, and (**E**) dehydrogenase activity. Values are expressed as mean ± standard deviation (SD) of three replicates (*n* = 3). Different lowercase letters above bars indicate statistically significant differences among treatments at *p* < 0.05.

Biochar amendment significantly affected all measured wheat growth parameters and root dehydrogenase activity (one-way ANOVA, *p* < 0.001). Zn-BC produced the highest shoot length, root length, biomass, leaf area, and root dehydrogenase activity, followed by BC and the untreated control (Table 6, Figure 6 and Figure 7). Tukey’s HSD test showed significant differences among the three treatments (*p* < 0.05), as indicated by different superscript letters in Table 6.

Soil pH increased from 5.4 ± 0.1 in the control to 6.2 ± 0.1 and 6.6 ± 0.1 following BC and Zn-BC application, respectively (Table 5). This increase was accompanied by an increase in CEC from 10.1 ± 0.5 cmol_c kg^−1^ in the control to 14.6 ± 0.7 and 18.3 ± 0.9 cmol_c kg^−1^ in BC- and Zn-BC-amended soils, respectively. However, considering the near-neutral pH of Zn-BC itself (6.8 ± 0.1), the observed soil pH increase cannot be attributed solely to a direct liming effect of the amendment. As carbonate content and individual exchangeable base cations were not quantified, the specific mechanism responsible for the observed pH increase could not be conclusively determined.

### 3.4. Soil Fertility and Health Assessment

Post-harvest analysis of soil revealed that the application of zinc-modified coconut shell biochar (Zn-BC) led to significant improvements in several key soil physicochemical and biological parameters compared to the untreated control. Soil pH increased from 5.4 to 6.6, reflecting the liming effect of the alkaline biochar and its buffering capacity in acidic, e-waste-contaminated soils. Electrical conductivity (EC) also increased moderately, indicating enhanced nutrient availability. Zn-BC application increased the measured soil NO_3_^−^-N and NH_4_^+^-N concentrations relative to the control (Table 7). However, the increase in EC should not be interpreted generally as enhanced nutrient availability, because EC reflects the overall concentration of soluble ions and does not distinguish between beneficial nutrient ions and potentially undesirable ions. Individual exchangeable Ca^2+^, Mg^2+^ and K^+^ and soluble Zn^2+^ and SO_4_^2−^ were not quantified in the present study; therefore, their respective contributions to the observed changes cannot be determined. Cation exchange capacity (CEC) was elevated from 10.1 to 18.3 cmol_c/kg, likely due to the increased surface area and functional groups introduced by biochar. Total organic carbon (TOC) and total nitrogen (TN) levels were also enhanced, indicating better organic matter accumulation and nutrient status. Biological indicators showed marked improvement; microbial biomass carbon (MBC) more than doubled, while dehydrogenase and phosphatase activities increased substantially, highlighting improved microbial activity increases in farmland soil organic carbon of this kind are recognized as an important indicator of soil quality recovery following anthropogenic disturbance [40], and the concurrent improvement in nitrogen status is consistent with the close coupling between soil organic carbon and nitrogen–phosphorus cycling reported in other soil-ecosystem studies [41] and enzymatic functioning in the amended soil. Overall, Zn-BC amendment significantly improved soil fertility and biological health under e-waste-induced stress conditions. The results are summarized in Table 7.

## 4. Discussion

Zn modification substantially altered the textural properties of coconut shell biochar, particularly its surface area and pore volume, while maintaining a mesoporous structure. The greater accessible surface and pore network of Zn-BC may provide additional sites for PAE retention and contribute to its greater adsorption performance relative to unmodified BC [42]. A comparable relationship between amendment porosity and contaminant transport restriction has been demonstrated for bio-based hydrogel-modified soils, where micro scale pore structure governed physical barriers limiting pollutant mobility [43].

The improved wheat growth observed under Zn-BC treatment was associated with greater PAE retention and improved soil physicochemical and biological conditions. Immobilization of DMP and DBP may reduce contaminant exposure and associated stress in the rhizosphere, while improvements in cation exchange capacity, organic carbon, and microbial activity may promote nutrient cycling and root development [44]. Thus, Zn-BC may provide complementary functions as both a contaminant sorbent and soil-quality amendment. Its adsorption performance was also greater than values previously reported for pristine agricultural biochars [45], supporting the potential benefit of Zn functionalization.

Several interactions may contribute to PAE retention by Zn-BC. Zn-containing surface phases may interact with ester carbonyl groups, while hydroxyl and other oxygen-containing functional groups identified by FTIR may contribute to hydrogen bonding [46]. Hydrophobic and π–π interactions between the carbonaceous surface and aromatic PAE structures may also contribute; however, these mechanisms were not directly quantified in the present study. Hydrophobic partitioning may be particularly relevant for the more hydrophobic DBP. Although the pseudo-second-order model provided the best mathematical description of the adsorption kinetics, kinetic-model fitting alone does not establish a specific adsorption mechanism. Direct surface analyses such as X-ray photoelectron spectroscopy (XPS), together with theoretical approaches such as density functional theory (DFT), would be required to resolve the relative contributions of these interactions.

The superior shoot and root development following Zn-BC application may reflect the combined effects of improved soil conditions and reduced PAE-associated phytotoxic stress. Zinc is involved in plant enzymatic functions, auxin biosynthesis, and protein metabolism [47], whereas biochar can improve soil physical properties, including porosity and water retention [48]. Their combined effects may therefore create more favorable conditions for root development and above-ground growth. Improvements in biomass and leaf development may similarly be associated with enhanced water and nutrient acquisition and reduced contaminant bioavailability. Metal-functionalized biochars have been reported to enhance the immobilization of organic pollutants, thereby limiting their potential toxicity to plants [49].

Dehydrogenase activity is an indicator of microbial oxidative metabolism and provides information on soil biological functioning. Its enhancement following biochar amendment, particularly with Zn-BC, may reflect more favorable physicochemical conditions and reduced contaminant stress in the rhizosphere. Soil biological responses are closely linked to vegetation and soil management; studies on constructed soils have similarly demonstrated that vegetation and time influence soil development, organic-matter dynamics, and enzymatic activity, including dehydrogenase activity [50]. The concurrent improvement in wheat performance and soil biological activity therefore suggests that Zn-BC affects both plant development and biological functioning of the soil. Enhanced phosphatase activity further indicates improved potential for phosphorus cycling. Biochar may also provide microsites and carbon resources that support microbial communities under environmental stress [51].

The overall improvement in wheat performance following BC, and particularly Zn-BC, is consistent with the combined influence of improved soil physicochemical conditions and reduced contaminant availability. Comparable soil–plant relationships have been reported following organic amendment application, where improved nutrient availability and soil quality were associated with enhanced plant physiological performance, although responses depended on plant species and exposure duration. More broadly, previous studies have demonstrated the capacity of modified biochars to immobilize both inorganic and organic contaminants while improving plant growth and soil quality [52]. The present findings extend these observations to DMP- and DBP-contaminated agricultural soil.

From a pedological perspective, PAE behavior may also be influenced by soil-forming processes and parent-material characteristics through their effects on organic matter, clay minerals, reactive surfaces, soil acidity, and exchange capacity [53]. However, the present experiment used homogenized composite soil from the 0–15 cm surface/root-zone interval rather than separately characterized genetic horizons. Consequently, amendment responses cannot be attributed to specific pedogenic horizons or horizon-forming processes. Future field investigations incorporating profile description and horizon-resolved sampling would help determine whether pedogenic differentiation influences the performance of BC and Zn-BC in PAE-contaminated soils [54].

Several limitations should be considered. The experiment was conducted under controlled conditions using composite soil and therefore may not fully represent field-scale spatial and environmental heterogeneity. Direct spectroscopic confirmation of post-adsorption interactions was not performed, and corresponding post-harvest soil fertility and biological measurements for the unmodified BC treatment were not separately quantified. In addition, chlorophyll content, SPAD index, and photosynthetic rate were not measured. Future studies should incorporate these physiological endpoints, direct surface characterization, and long-term field experiments to evaluate the stability and environmental performance of Zn-BC under realistic agricultural conditions.

From an environmental-management perspective, Zn-modified biochar represents a potentially useful approach for PAE-contaminated agricultural soils because contaminant immobilization can be coupled with improvements in soil physicochemical and biological functioning. Field validation and assessment of long-term amendment stability will nevertheless be necessary before practical application.

## 5. Conclusions

The present study confirms the potential of zinc-modified coconut shell biochar (Zn-BC) as an effective soil amendment for improving wheat growth in phthalate-contaminated soils. Zn-BC significantly enhanced key growth parameters and soil enzymatic activity compared to both unmodified biochar and untreated controls. The synergistic effects of zinc and biochar not only improved plant physiological traits but also likely contributed to the detoxification of soil through pollutant immobilization and stimulation of microbial function.

These findings suggest that Zn-BC could serve as a promising strategy for remediating e-waste-polluted agricultural lands while supporting crop productivity. Further research, including field-scale validation and long-term environmental impact assessments, is recommended to support practical implementation.

Although direct spectroscopic evidence (e.g., XPS, FTIR after adsorption) was not obtained in this study, the combined characterization and kinetic data suggest that Zn-BC immobilizes DMP and DBP primarily through surface complexation, hydrogen bonding, π-π EDA interactions, and hydrophobic partitioning. Further investigations employing advanced spectroscopic and theoretical modeling approaches are warranted to resolve the precise molecular mechanisms.

From a regional and global soil-ecological perspective, these findings are particularly relevant for agricultural soils located near e-waste recycling and urban-industrial zones, where phthalate contamination threatens soil quality, crop safety, and long-term agricultural sustainability. The observed improvements in nutrient retention, microbial biomass, and enzymatic activity suggest that Zn-modified biochar may contribute not only to contaminant immobilization but also to restoration of soil-ecosystem functioning in degraded agroecosystems. Given the increasing global concern regarding organic contaminants in food-production systems, engineered biochar amendments may represent a sustainable strategy for maintaining soil fertility and reducing contaminant transfer into the food chain.

## Figures and Tables

**Table 1:** Soil samples collected from wheat root area (0–15 cm) in Islamabad and Rawalpindi.

S. No	Samples ID	Location of Samples	No of Sub-Samples	Latitude (° N)	Longitude (° E)
1	KP	Khanpur	5	33.80	72.93
2	BG	Bani gala	5	33.71	73.154
3	AP	Alipur	5	33.64	73.178
4	QF	Qasim farm	5	33.72	72.949
5	NCRD	NCRD	5	33.69	73.131
6	SF	Shehzad Farm	5	33.67	73.14
7	SC	Sanam Chowk	5	33.682	73.145
8	D	Dokazak	5	33.73	73.056
9	CS	Commodore School	5	33.69	73.131
10	FT	Farash Town	5	33.651	73.204
11	IC	Islamabad, Chak Shahzad	5	33.690	73.131
12	IG	Islamabad, Golra	5	33.695	72.977
13	IKK	Islamabad, Khyaban-e-Kashmir	5	33.635	72.922
14	R	Rawalpindi road	5	33.68	72.80

Note: All samples were composited in equal proportions to create a homogenous soil mixture for the pot experiment.

**Table 2:** Initial physiochemical properties of the composite e-waste-contaminated soil used in the pot experiment.

Parameter	Value	Method
pH (1:5 soil:water)	5.4 ± 0.2	pH meter
Electrical conductivity (µS/cm)	350 ± 20	EC meter
Total organic carbon (TOC, %)	0.62 ± 0.07	Walkley Black method
Total nitrogen (TN, %)	0.07 ± 0.01	Kjeldahl method
Cation exchange capacity (CEC, cmolc/kg)	10.1 ± 0.8	Ammonium acetate method
Soil Texture	Sandy Loam	Hydrometer method
Sand (%)	68 ± 3	–
Silt (%)	20 ± 2	–
Clay (%)	12 ± 2	–
DMP concentration (mg/kg)	12.4 ± 1.8	GC-MS after extraction
DBP concentration (mg/kg)	18.7 ± 2.3	GC-MS after extraction
Total PAEs (mg/kg)	31.1 ± 3.5	–

Note: Values represent mean ± standard deviation (*n* = 3). DMP and DBP were quantified using gas chromatography–mass spectrometry (GC-MS) following ultrasonic extraction with dichloromethane (USEPA Method 3550C). “–” indicates not applicable.

**Table 3:** Physicochemical and textural properties of BC and Zn-BC.

Property	BC	Zn-BC	Change (%)
pH (1:10)	6.5 ± 0.1 ^b^	6.8 ± 0.1 ^a^	↑ 4.6%
EC (µS/cm)	2.1 ± 0.2 ^b^	2.6 ± 0.2 ^a^	↑ 23.8%
Yield (%)	28.0 ± 1.5 ^a^	26.5 ± 1.2 ^a^	↓ 5.4%
Moisture content (%)	2.06 ± 0.1 ^a^	1.95 ± 0.1 ^a^	↓ 5.3%
Ash content (%)	7.5 ± 0.3 ^b^	8.2 ± 0.3 ^a^	↑ 9.3%
BET surface area (m^2^/g)	185.4 ± 5.2 ^b^	267.8 ± 6.1 ^a^	↑ 44.4%
Total pore volume (cm^3^/g)	0.12 ± 0.01 ^b^	0.19 ± 0.01 ^a^	↑ 58.3%
Average pore diameter (nm)	3.8 ± 0.2 ^a^	3.6 ± 0.2 ^a^	↓ 5.3%

Note: Means within a row followed by different superscript letters differ significantly between BC and Zn-BC (Welch’s two-sample *t*-test, *p* < 0.05, *n* = 3 independent syntheses per material). ↑ indicates an increase and ↓ indicates a decrease in Zn-BC relative to BC. Percentage change was calculated relative to BC.

**Table 4:** Elemental composition (XRF) of BC and Zn-BC (ppm).

Element	BC (ppm)	Zn-BC (ppm)	Change (%)
Zn	120 ± 15 ^b^	8138 ± 120 ^a^	↑ 6682%
Fe	5140 ± 80 ^b^	6305 ± 95 ^a^	↑ 22.6%
Mn	1200 ± 30 ^b^	1500 ± 40 ^a^	↑ 25.0%
K	18,500 ± 200 ^b^	19,700 ± 180 ^a^	↑ 6.5%
Ca	12,400 ± 150 ^a^	11,200 ± 140 ^b^	↓ 9.7%
Mg	3200 ± 50 ^b^	3450 ± 55 ^a^	↑ 7.8%
P	1800 ± 40 ^b^	1950 ± 45 ^a^	↑ 8.3%

Note: Means within a row followed by different superscript letters differ significantly between BC and Zn-BC (Welch’s two-sample *t*-test, *p* < 0.05, *n* = 3 independent syntheses per material). ↑ indicates an increase and ↓ indicates a decrease in Zn-BC relative to BC. Percentage change was calculated relative to BC.

**Table 5:** Degradation experiment of DMP and DBP with biochar and zinc-modified biochar.

Time (min)	DMP (BC)	DMP (Zn-BC)	DBP (BC)	DBP (Zn-BC)
0	0.00 ± 0.00	0.00 ± 0.00	0.00 ± 0.00	0.00 ± 0.00
5	9.97 ± 0.06	12.00 ± 0.00	6.00 ± 0.00	8.00 ± 0.00
15	16.00 ± 0.00	18.03 ± 0.06	12.00 ± 0.00	14.00 ± 0.00
30	18.17 ± 0.29	20.00 ± 0.00	16.00 ± 0.00	18.00 ± 0.00
60	19.37 ± 0.35	21.33 ± 0.29	18.17 ± 0.29	21.33 ± 0.29
90	20.20 ± 0.00	22.17 ± 0.29	19.17 ± 0.29	23.17 ± 0.29
120	20.33 ± 0.29	23.00 ± 0.00	20.00 ± 0.00	24.00 ± 0.00

**Table 6:** Effects of control, biochar (BC), and zinc-modified biochar (Zn-BC) on wheat growth parameters (shoot height, root length, biomass, and leaf area) and root dehydrogenase activity.

Parameter	Control (Mean ± SD)	BC (Mean ± SD)	Zn-BC (Mean ± SD)
Shoot length (cm)	17.40 ± 0.10 ^c^	23.20 ± 0.10 ^b^	29.43 ± 2.70 ^a^
Root length (cm)	7.23 ± 0.25 ^c^	11.70 ± 0.30 ^b^	15.50 ± 0.36 ^a^
Biomass (g)	6.70 ± 0.20 ^c^	9.90 ± 0.26 ^b^	12.80 ± 0.35 ^a^
Leaf area (cm^2^)	1970 ± 10 ^c^	3100 ± 10 ^b^	3550 ± 40.3 ^a^
Root dehydrogenase activity	0.76 ± 0.03 ^c^	1.25 ± 0.09 ^b^	2.23 ± 0.06 ^a^

Note: Means within a row followed by different superscript letters differ significantly among CK, BC, and Zn-BC (one-way ANOVA followed by Tukey’s HSD post hoc test, *p* < 0.05, *n* = 3 biological replicates per treatment).

**Table 7:** Post-harvest soil fertility and biological indicators in untreated soil and soil treated with Zn-modified biochar (Zn-BC).

Parameter	Untreated Soil	Treated Soil (Zn-BC, After Wheat)
pH (1:5)	5.4 ± 0.2 ^b^	6.6 ± 0.1 ^a^
EC (µS/cm)	350 ± 20 ^b^	510 ± 30 ^a^
NO_3_^−^-N (mg/kg)	12.5 ± 2.0 ^b^	22.0 ± 3.0 ^a^
NH_4_^+^-N (mg/kg)	8.2 ± 1.5 ^b^	17.4 ± 2.2 ^a^
CEC (cmolc/kg)	10.1 ± 0.8 ^b^	18.3 ± 1.2 ^a^
TOC (%)	0.62 ± 0.07 ^b^	1.85 ± 0.12 ^a^
Total Nitrogen (TN) (%)	0.07 ± 0.01 ^b^	0.15 ± 0.02 ^a^
Microbial Biomass C (mg/kg)	130 ± 10 ^b^	320 ± 25 ^a^
Dehydrogenase Activity (µg TPF/g/d)	16.5 ± 2.0 ^b^	45.2 ± 4.0 ^a^
Phosphatase Activity (µg PNP/g/h)	21.4 ± 2.5 ^b^	58.6 ± 5.0 ^a^

Note: Means within a row followed by different superscript letters differ significantly between untreated and Zn-BC-treated soil (Welch’s two-sample *t*-test, *p* < 0.05, *n* = 3 biological replicates per treatment). Post-harvest soil fertility and biological indicators were assessed for the untreated control and the top-performing Zn-BC treatment; corresponding data for the unmodified BC treatment were not separately quantified in this study, which is noted as a limitation in the Discussion.

## Data Availability

The raw data supporting the conclusions of this article will be made available by the authors on request.
